# Pioglitazone for NAFLD Patients With Prediabetes or Type 2 Diabetes Mellitus: A Meta-Analysis

**DOI:** 10.3389/fendo.2021.615409

**Published:** 2021-04-28

**Authors:** Jingxuan Lian, Jianfang Fu

**Affiliations:** Department of Endocrinology, Xijing Hospital of Air Force Medical University, Xi’an, China

**Keywords:** NAFLD, type 2 diabetes mellitus, pioglitazone, meta-analysis, prediabetes

## Abstract

**Objective:**

To systematically evaluate the effects of pioglitazone in the treatment of patients with prediabetes or T2DM combined with NAFLD.

**Methods:**

The Cochrane Central Register of Controlled Trials (CENTRAL), Embase, and ClinicalTrials databases were searched until August 2020 for publications written in English. Two reviewers independently assessed study eligibility, continuous data extraction, independent assessment of bias risk, and graded the strength of evidence. Our primary outcomes were the individual number of patients with improvement of at least 1 point in each of the histological parameters. Baseline characteristic data, such as BMI, weight, total body fat, fasting plasma glucose and fasting plasma insulin, and liver biological indicators, such as triglyceride level, HDL cholesterol level, plasma AST, and plasma ALT, were used as secondary outcomes.

**Results:**

A total of 4 studies were included. Compared with placebo, pioglitazone significantly improved steatosis grade, inflammation grade and ballooning grade, while in the fibrosis stage, there was no significant improvement in pioglitazone compared with placebo. In addition, pioglitazone can also improve blood glucose and liver function.

**Conclusion:**

Pioglitazone can significantly improve the histological performance of the liver and insulin sensitivity. Additionally, it can significantly reduce fasting blood glucose, glycosylated hemoglobin, plasma AST, ALT and other liver biological indicators. Due to the lack of relevant randomized controlled trials and short intervention times, long-term studies are still needed to verify its efficacy and safety.

**Systematic Review Registration:**

[PROSPERO], identifier [CRD42020212025].

## Introduction

Nonalcoholic fatty liver disease (NAFLD) is a liver disease caused by damage to the liver unrelated to alcohol; NAFLD can develop into nonalcoholic steatosis (NASH), advanced fibrosis and hepatocellular carcinoma (HCC) (1). NAFLD is the fastest growing cause of HCC among liver transplantation candidates (2). NAFLD may also be involved in the pathogenesis of type 2 diabetes and atherosclerosis. Researchers recently reported that the global prevalence of NAFLD in T2DM patients was 55.5% (3).

The European Association for the Study of Obesity recommends in the NAFLD Clinical Practice Guidelines that screening for NAFLD in T2DM patients, although weight reduction is the basis for the treatment of NAFLD, drug therapy is also necessary because weight reduction alone is difficult to maintain therapeutic effects (4). In patients with T2DM complicated with NAFLD, the use of antidiabetic drugs is more effective than lifestyle changes in controlling glucose levels (5). In addition, lifestyle changes, combined with antidiabetic drugs, may reduce cardiovascular disease-related risk factors and liver fat accumulation and delay the progression of NASH and fibrosis. While there is no recognized drug for the treatment of NAFLD, many antidiabetic drugs have been tested in NAFLD patients due to the common epidemiological and pathophysiological characteristics between NAFLD and T2DM (6).

Pioglitazone, a thiazolidinedione derivative, is a peroxisome proliferator–activated receptor γ (PPARγ) agonist that ameliorates insulin resistance and improves glucose and lipid metabolism in type 2 diabetes (7, 8). Although a number of randomized controlled trials have been conducted to study the efficacy of pioglitazone in NAFLD combined with T2DM or prediabetes, there is no systematic review on randomized controlled trials (RCTs) of pioglitazone. Therefore, through the latest randomized controlled trials, we systematically reviewed the advantages and disadvantages of pioglitazone in the treatment of patients with prediabetes or T2DM combined with NAFLD.

## Methods

### Data Sources and Search Strategy

The systematic review scheme used in this study is reported in accordance with the preferred reporting items listed in the guidelines for systematic review and meta-analysis (PRISMA-P) (9). Our PROSPERO registration ID is CRD42020212025.

We performed electronic searches in the following databases: Cochrane Central Register of Controlled Trials (CENTRAL), Embase, and ClinicalTrials. We searched each database until August 2020, and the language was limited to English (the complete search strategy is shown in Appendix 1). The preferred reporting items of the systematic review and meta-analysis guidelines were followed at all stages of the study (the complete protocol is shown in Appendix 2).

### Inclusion and Exclusion Criteria

We included a double-blind, placebo-controlled RCT of the effects of pioglitazone on patients aged between 18 and 70 with prediabetes or T2DM combined with NAFLD. Intervention duration < 4 weeks or any cause of chronic liver disease other than NAFLD and suffering from other types of chronic diseases, such as hypertension and coronary heart disease, were excluded, and studies that did not differentiate patients in the subgroup analysis were excluded. In addition, long-term use of hypoglycemic drugs, prescription drugs for the treatment of other chronic diseases, food supplements, and other nonhyperglycemic drugs (such as liver protection drugs) were excluded. For the crossover RCTs, the carrying effect is considered; therefore, we used the data from the first phase of the study.

### Study Selection and Data Extraction

Two reviewers independently screened and identified the study and resolved their differences through discussion. In addition, a manual search of references in published systematic reviews and meta-analyses was performed to ensure that no studies were missing. Data were independently extracted to an Excel spreadsheet according to previously defined criteria. For each of the included studies, we extracted data such as study time, trial design, intervention measures and time, demographics, and baseline characteristics.

Our primary outcomes were the individual number of patients with improvement of at least 1 point in each of the histological parameters, which included four aspects: steatosis grade, inflammation grade, ballooning grade and fibrosis stage. Second, it includes resolution of NASH without worsening of fibrosis and individual histological score number of patients with improvement of at least 2 points from two different parameters. Our secondary outcomes were baseline characteristic data, such as BMI, weight, total body fat, fasting plasma glucose and fasting plasma insulin, and liver biological indicators, such as triglyceride level, HDL cholesterol level, plasma AST, and plasma ALT.

### Quality Assessment

The Cochrane collaborative risk assessment tool was used to assess the risk of bias for each included study. The following aspects were evaluated: random sequence generation, allocation hiding, blindness of participants and personnel, blindness of result evaluation, incomplete result data, selective reporting and other biases. GRADE Pro (V.3.6) software was used to rate the overall quality of evidence for each outcome based on five evaluation criteria: risk of bias, inconsistency, indirectness, imprecision, and publication bias.

### Data Synthesis and Statistical Analysis

For continuity variables, we used the standardized mean difference (SMD) and 95% CI for analysis. The meanings of SMD values should be interpreted as follows: 0.2 represents a small effect, 0.5 a moderate effect, and 0.8 a large effect. For dichotomous variables, we calculated the risk ratio of the 95% CI. We used changes before and after the intervention to assess the effectiveness of different drugs and placebos. When SD was not reported in the study, we derived SD from other studies included in the meta-analysis. We used P=0.05 as the statistically significant threshold. Meta-analysis software (RevMan V.5.3) was used for analysis, and heterogeneity was evaluated according to I2: 25%, 50 and 75% values were judged as mild, moderate and substantial heterogeneity, respectively. The random effects model was used to reduce the effect of heterogeneity. GRADE Pro (V.3.6) software was used to rate the overall quality of evidence for each outcome based on five evaluation criteria: risk of bias, inconsistency, indirectness, imprecision, and publication bias.

## Results

**Figure 1:**
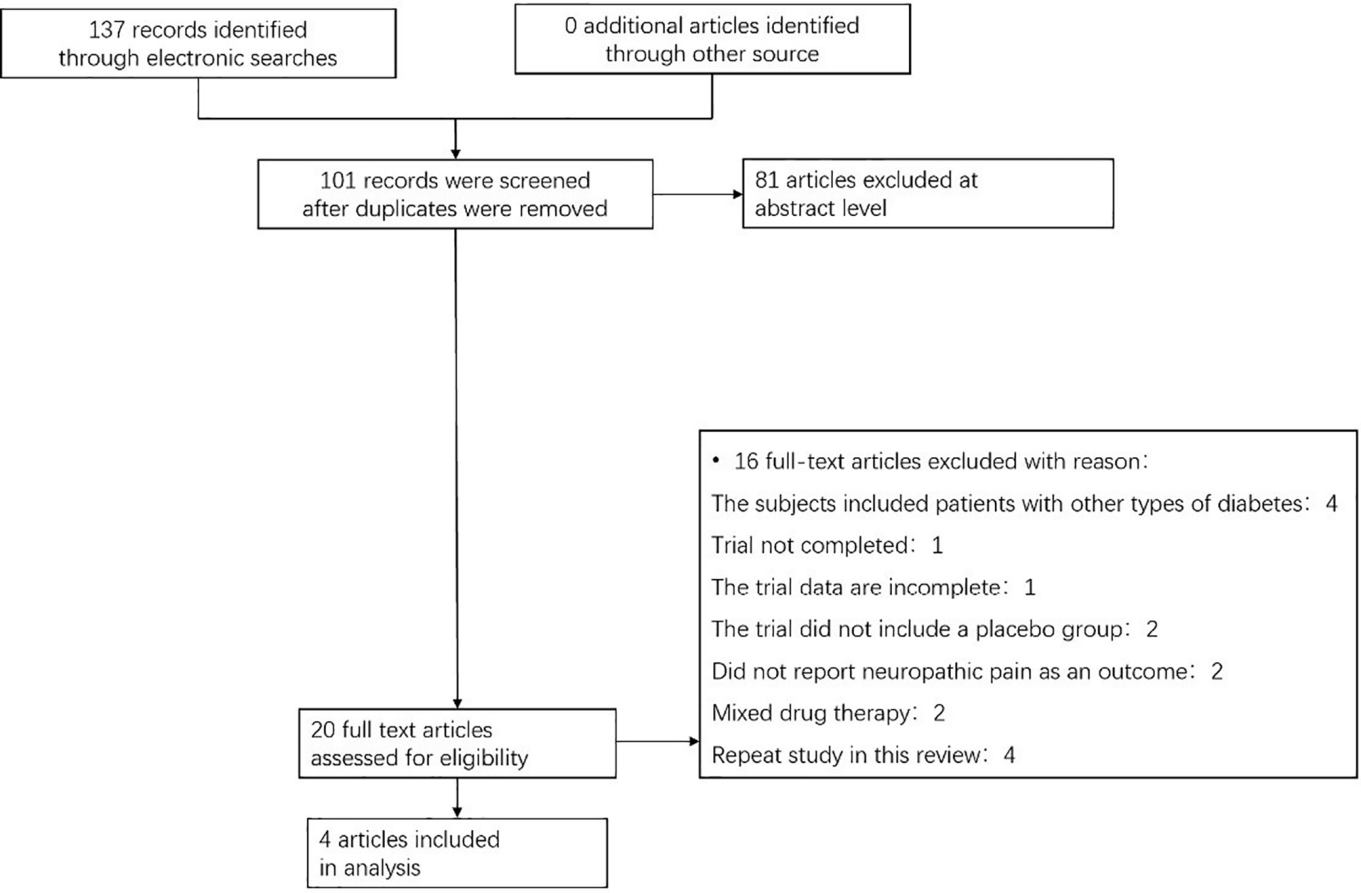
Flow chart showing the process for inclusion of RCTs about patients with prediabetes or T2DM combined with NAFLD. RCTs, randomized clinical trials.

We found a total of 137 articles from the target database, among which 138 full-text papers were qualified, and 20 full-text papers were excluded according to the research exclusion criteria (**Figure 1**). A total of 4 RCTs of pioglitazone in the treatment of patients with prediabetes or T2DM combined with NAFLD met the inclusion criteria (9–12). Most of the trials were conducted in the United States, including one in China, where the intervention duration was more than four months, with a maximum of 18 months.

**Figure 2:**
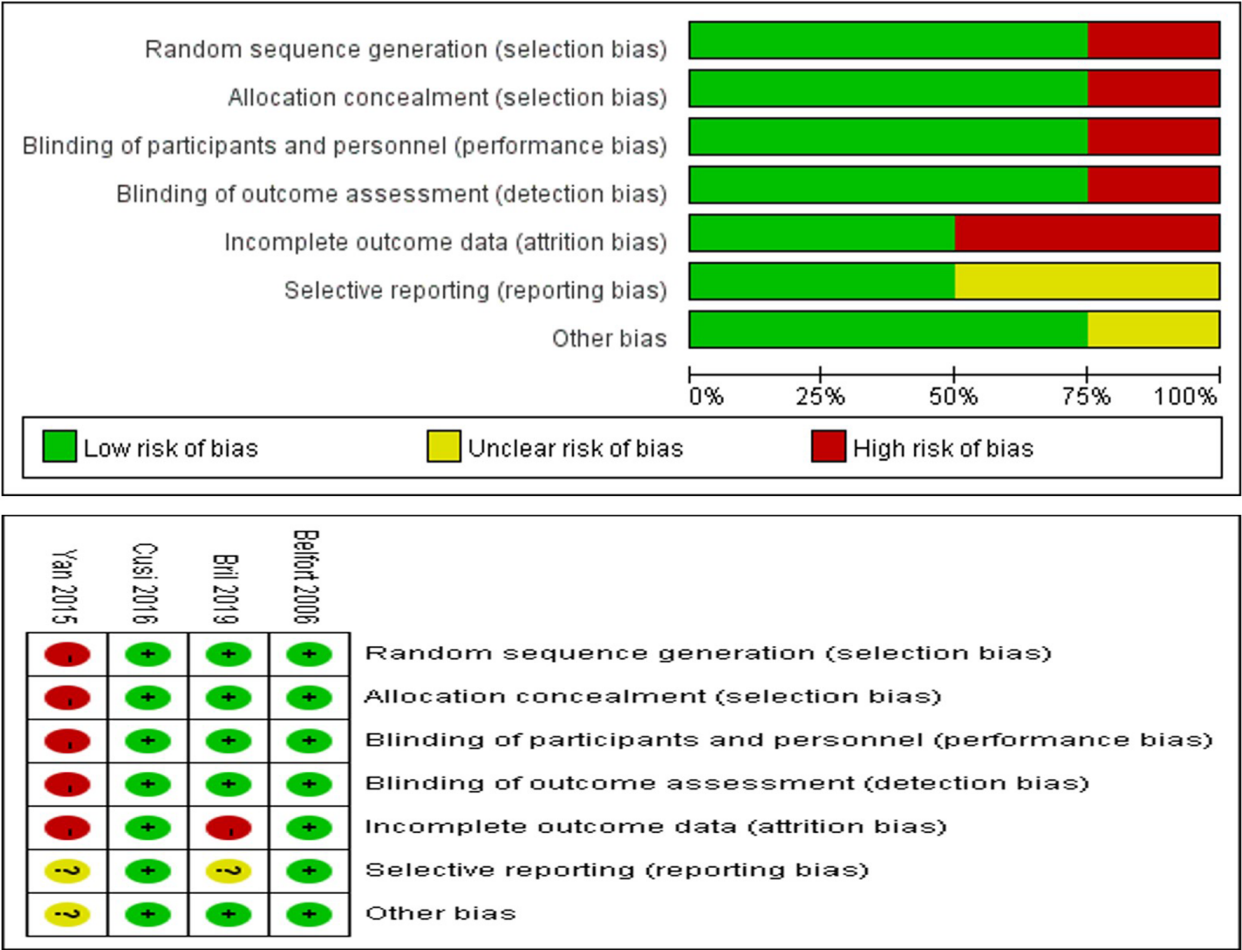
Risk of bias summary for review authors’ judgments about each risk of bias item for each included study.

Through the risk assessment using the Cochrane Collaboration risk assessment tool, it was found that the overall risk was moderate to low (**Figure 2**), which was mainly caused by allocation concealment, selective reporting, incomplete data, and unclear blind reporting in some studies.

### Primary Outcomes

**Figure 3:**
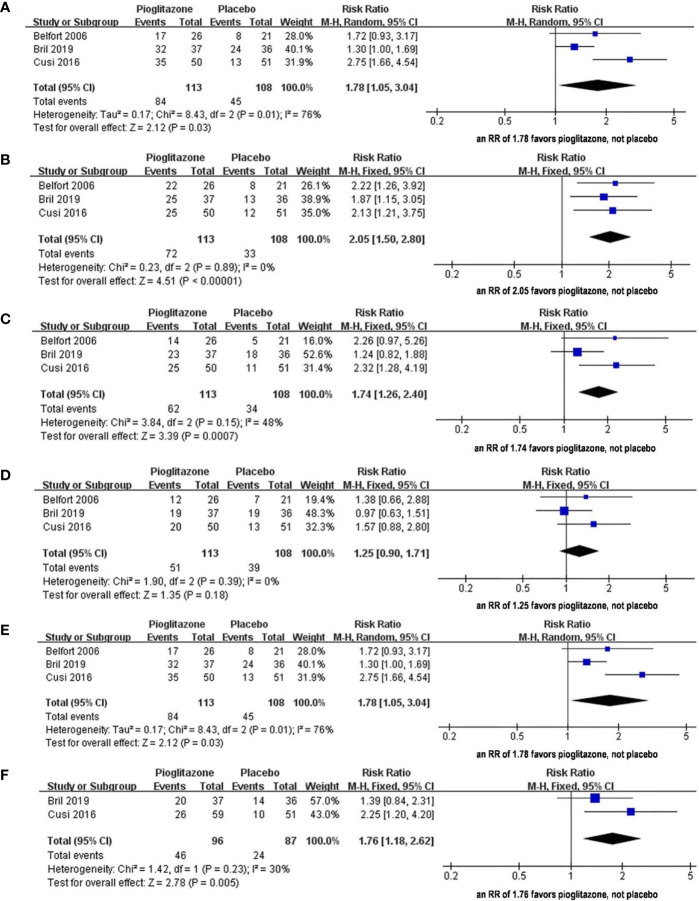
The effect of pioglitazone in hepatic histologic scores(steatosis grade) with improvement of at least 1 grade **(A)**; The effect of pioglitazone in hepatic histologic scores(Inflammation grade) with improvement of at least 1 grade **(B)**; The effect of pioglitazone in hepatic histologic scores(ballooning grade) with improvement of at least 1 grade **(C)**; The effect of pioglitazone in hepatic histologic scores(fibrosis stage) with improvement of at least 1 grade **(D)**; The effect of pioglitazone in resolution of NASH without worsening of fibrosis **(E)**; The effect of pioglitazone in reduction of at least 2 points in hepatic histologic scores (from two different parameters) **(F)**.

The meta-analysis showed (**Figure 3**) that in terms of the liver histological score, compared with placebo, pioglitazone significantly improved the steatosis grade [RR 1.78 (95% CI 1.05, 3.04, p= 0.03, I^2^ = 76%)], and the quality of the evidence was very low [summary of findings (SoF) **Table 1**]. There were also significant differences in inflammation grade [RR2.05 (95% CI 1.50, 2.80, p<0.00001, I^2^ = 0%; SoF **Table 1**)] and ballooning grade [RR 1.74 (95% CI 1.26, 2.40, p= 0.0007, I^2^ = 48%; SoF **Table 1**)]. However, in the fibrosis stage (**Figure 3D**), there was no significant improvement in pioglitazone compared with placebo [RR 1.25 (95% CI 0.90, 1.71, p= 0.18, I^2^ = 48%; SoF **Table 1**)]. In terms of the resolution of NASH (**Figure 3E**), compared with placebo, pioglitazone also significantly improved the rate of NASH resolution [RR1.76 (95% CI 1.18, 2.62, p= 0.005, I^2^ = 30%; SoF **Table 1**)]. In terms of individual histological scores, the number of patients with improvement of at least 2 points from two different parameters (**Figure 3F**) was also significantly higher in the pioglitazone group than in the placebo group [RR2.35 (95% CI 1.29, 4.29, P = 0.005, I^2^ = 55%; SoF **Table 1**)].

**Table 1:** Pioglitazone compared to Placebo for NAFLD Patients with Prediabetes or Type 2 Diabetes Mellitus.

Patient or population: NAFLD Patients with Prediabetes or Type 2 Diabetes Mellitus Settings: Intervention, Pioglitazone, Comparison, Placebo						
Outcomes	Illustrative comparative risks* (95% CI)		Relative effect(95% CI)	No of Participants(studies)	Quality of the evidence(GRADE)	Comments
	Assumed risk	Corresponding risk				
	Placebo	Pioglitazone				
**Hepatic Histologic Scores with improvement of at least 1 grade (Steatosis grade)**	**Study population**		**RR 1.78** (1.05 to 3.04)	221 (3 studies)	⊕⊝⊝⊝ **very low** ^1,2,3^	
	**417 per 1000**	**742 per 1000** (437 to 1000)				
	**Moderate**					
	**381 per 1000**	**678 per 1000** (400 to 1000)				
**Hepatic Histologic Scores with improvement of at least 1 grade (Inflammation grade)**	**Study population**		**RR 2.05** (1.5 to 2.8)	221 (3 studies)	⊕⊕⊕⊝ **moderate** ^2,3,4^	
	**306 per 1000**	**626 per 1000** (458 to 856)				
	**Moderate**					
	**361 per 1000**	**740 per 1000** (541 to 1000)				
**Hepatic Histologic Scores with improvement of at least 1 grade (Ballooning grade)**	**Study population**		**RR 1.74** (1.26 to 2.4)	221 (3 studies)	⊕⊝⊝⊝ **very low** ^1,2,3^	
	**315 per 1000**	**548 per 1000** (397 to 756)				
	**Moderate**					
	**238 per 1000**	**414 per 1000** (300 to 571)				
**Hepatic Histologic Scores with improvement of at least 1 grade (Fibrosis stage)**	**Study population**		**RR 1.25** (0.9 to 1.71)	221 (3 studies)	⊕⊕⊝⊝ **low** ^2,3^	
	**361 per 1000**	**451 per 1000** (325 to 618)				
	**Moderate**					
	**333 per 1000**	**416 per 1000** (300 to 569)				
**Resolution of NASH without worsening of fibrosis**	**Study population**		**RR 1.76** (1.18 to 2.62)	183 (2 studies)	⊕⊝⊝⊝ **very low** ^2,3,5^	
	**276 per 1000**	**486 per 1000** (326 to 723)				
	**Moderate**					
	**293 per 1000**	**516 per 1000** (346 to 768)				
**reduction of at least 2 points in Hepatic Histologic Scores Hepatic (from two different parameters)**	**Study population**		**RR 2.35** (1.29 to 4.29)	174 (2 studies)	⊕⊕⊝⊝ **low** ^1,2,3,4^	
	**253 per 1000**	**594 per 1000** (326 to 1000)				
	**Moderate**					
	**269 per 1000**	**632 per 1000** (347 to 1000)				

GRADE Working Group grades of evidence.

**High quality:** Further research is very unlikely to change our confidence in the estimate of effect.

**Moderate quality:** Further research is likely to have an important impact on our confidence in the estimate of effect and may change the estimate.

**Low quality:** Further research is very likely to have an important impact on our confidence in the estimate of effect and is likely to change the estimate.

**Very low quality:** We are very uncertain about the estimate.

^1^Substantial heterogeneity.

^2^Some of the studies had adjuvant therapeutic measures, which to some extent led to indirect comparisons.

^3^Subjects which the study include was insufficient.

^4^RR > 2.

^5^Moderate heterogeneity.

### Basic Characteristics

**Figure 4:**
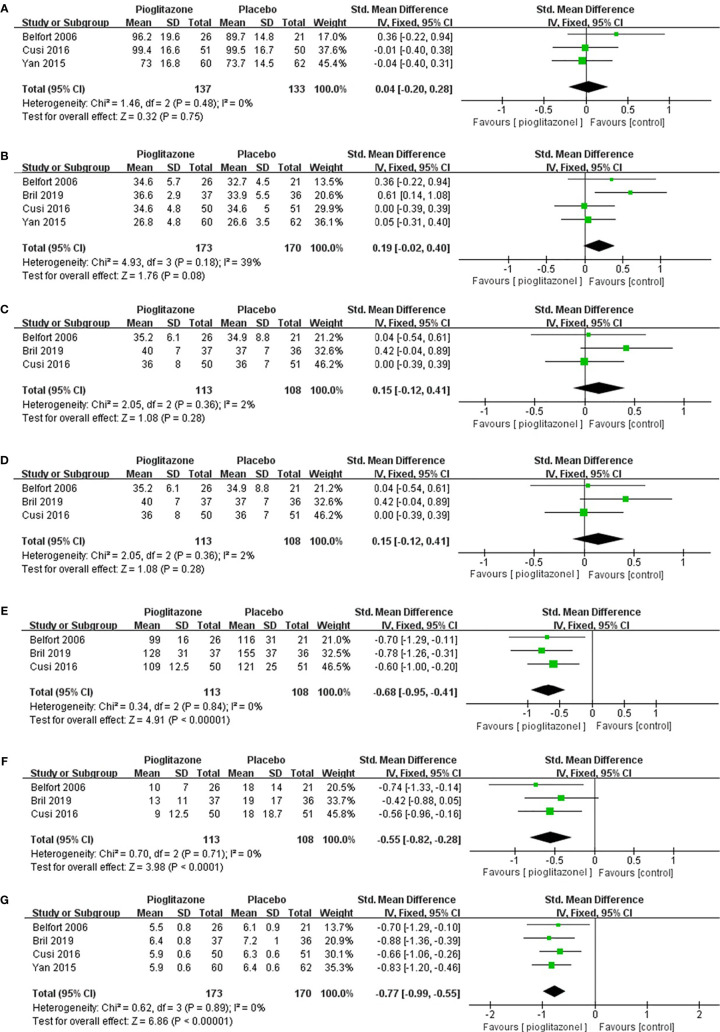
The effect of pioglitazone on weight **(A)**; the effect of pioglitazone on BMI **(B)**; the effect of pioglitazone on total body fat **(C)**; the effect of pioglitazone on HOMA-IR **(D)**; the effect of pioglitazone on fasting plasma glucose **(E)**; the effect of pioglitazone on fasting plasma insulin **(F)**; and the effect of pioglitazone on HbA1c **(G)**.

In terms of weight, although pioglitazone was found to increase the weight of patients in some studies, the meta-analysis showed that pioglitazone did not significantly increase the weight (**Figure 4A**) of patients compared with placebo (SMD0.04 [95% CI -0.20, 0.28, p= 0.75, I^2^ = 0%)] or BMI [SMD0.19 (95% CI -0.02, 0.40, p= 0.08, I^2^ = 39%; **Figure 4B**)]. For total body fat, the meta-analysis showed no significant difference between placebo and pioglitazone [SMD0.15 (95% CI -0.12, 0.41, p= 0.28, I^2^ = 2%; **Figure 4C**)]. In regard to glucose metabolism, only in HOMA-IR, pioglitazone was not significantly different from placebo [SMD -0.44 (95%CI -1.04, 0.15,p= 0.14, I^2^ = 79%; **Figure 4D**)], In addition, in fasting plasma glucose (SMD -0.68 [95%CI -0.95, -0.41, p<0.00001, I^2^ = 0%; **Figure 4E**)], fasting plasma insulin [SMD -0.55 (95%CI -0.82, -0.28, p<0.00001, I^2^ = 0%; **Figure 4F**)] and HbA1c [SMD -0.77(95%CI -0.99 -0.55, p<0.00001, I^2^ = 0%; **Figure 4G**)], pioglitazone was significantly lower than the placebo group, indicating that pioglitazone can significantly improve the glucose metabolic function of patients.

### Biological Indicators of Liver

**Figure 5:**
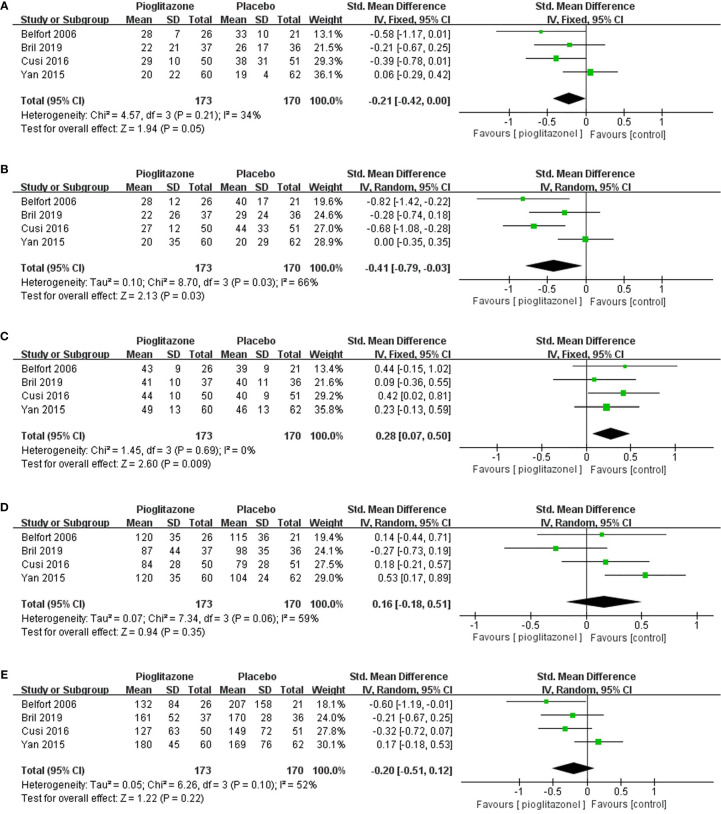
The effect of pioglitazone in plasma AST **(A)**; the effect of pioglitazone in plasma ALT **(B)**; the effect of pioglitazone in HDL **(C)**; the effect of pioglitazone in LDL **(D)**; and the effect of pioglitazone on triglyceride levels **(E)**.

We also analyzed other biomarkers of the liver, and meta-analysis showed that compared with placebo, pioglitazone could significantly reduce the plasma AST (SMD-0.21 [95% CI -0.42, 0.00, p= 0.05, I^2^ = 34%; **Figure 5A**)] and ALT [SMD -0.36 (95% CI-0.57, -0.14, p= 0.03, I^2^ = 66%; **Figure 5B**)] levels in patients and significantly improve the liver function of patients. Pioglitazone also significantly increased patients’ HDL levels [SMD 0.28 (95%CI 0.07, 0.50, p= 0.009, I^2^ = 0%; **Figure 5C**)], but there was no significant change in LDL levels compared with placebo [SMD 0.16 (95%CI -0.18, 0.51, p= 0.35, I^2^ = 59%; **Figure 5D**)]. Moreover, there was no significant difference in triglyceride levels between the pioglitazone group and the placebo group [SMD -0.20 (95% CI -0.51, 0.12, p= 0.22, I2 = 52%; **Figure 5E**)].

### Adverse Events

**Figure 6:**
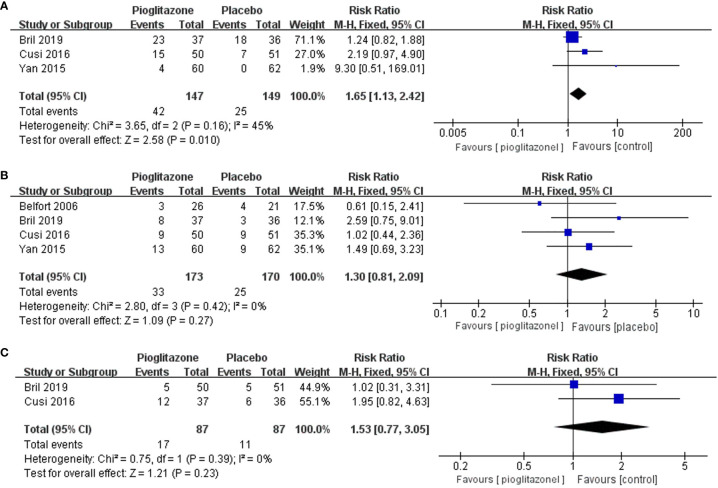
Effects of pioglitazone on the risk of adverse events in patients with pre-diabetes or T2DM combined with NAFLD **(A)**; Effects of pioglitazone on the risk of discontinuations in patients with pre-diabetes or T2DM combined with NAFLD **(B)**; Effects of pioglitazone on the risk of Serious Adverse Events in patients with pre-diabetes or T2DM combined with NAFLD **(C)**.

Meta-analysis showed that pioglitazone was significantly more likely to cause adverse events than placebo [RR 1.65 (95% CI 1.13, 2.42, p= 0.01, I2 = 45%; **Figure 6A**)]. The risk of experiencing individual adverse events of hypoglycemia, chronic lower limb edema, atypical chest pain or epigastralgia and back or joint pain was significantly increased with pioglitazone compared with placebo. However, there was no significant increase in the risk of pioglitazone discontinuation due to adverse events compared to placebo [RR 1.30 (95% CI 0.81, 2.09, p= 0.27, I2 = 0%; **Figure 6B**)].

There was also no significant difference in the risk of serious adverse events between pioglitazone and placebo [RR 1.53 (95% CI 0.77, 3.05, p= 0.23, I^2^ = 0%; **Figure 6C**)]. In total, four deaths were reported across 1 trial, two in the pioglitazone group and two in the placebo group.

### Publication Bias

**Figure 7:**
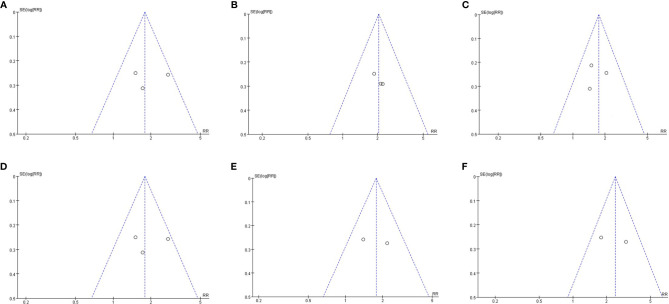
Publication bias assessment. **(A)** Funnel plots for hepatic histologic scores(steatosis grade); **(B)** Funnel plots for hepatic histologic scores(Inflammation grade); **(C)** Funnel plots for hepatic histologic scores(ballooning grade); **(D)** Funnel plots for hepatic histologic scores(fibrosis stage); Funnel plots for resolution of NASH **(E)**; Funnel plots for reduction of at least 2 points in hepatic histologic scores **(F)**.

Since we included fewer than 10 articles, publication bias testing was not recommended according to the Cochrane Collaboration Handbook. However, to better evaluate our research results, we performed a publication bias analysis on the primary outcomes by funnel plots. The visual inspection of funnel plots of our primary outcomes did not suggest potential publication bias (**Figure 7**).

For our primary outcomes, sensitivity analysis was used to assess the impact of each individual study on the pooled results by sequentially remove each eligible study. Deletion of any study has no significant effect on the results, indicating that the results were statistically stable and reliable.

## Discussion

Nonalcoholic fatty liver disease is a frequently neglected and undertreated condition among patients with T2DM. Recent studies indicate that most obese patients with T2DM have NAFLD on imaging (13–15). T2DM patients with NAFLD have higher all-cause, cardiovascular (CV), and liver-related mortality (3, 16). A 150-month follow-up study showed that T2DM doubled the risk of all-cause, liver-related death in NAFLD patients (17). Therefore, it is necessary to actively explore the treatment plan for T2DM patients with NAFLD.

The results of our meta-analysis show that the evidence from published RCTs suggests that pioglitazone could significantly improve glucose metabolism, liver function and liver histology, such as steatosis grade, inflammation grade and ballooning grade, although there was no significant difference in fibrosis between pioglitazone and placebo. However, our results show that pioglitazone has efficacy in patients with T2DM combined with NFALD.

NASH is a more serious and progressive form of NAFLD and is characterized by inflammation and hepatocyte ballooning that set off fibrosis and pose a risk of developing cirrhosis and hepatocellular carcinoma (18, 19). In addition, studies through liver biopsy have found that approximately 30% to 50% of patients have steatohepatitis even in the presence of normal plasma aminotransferase levels (20–22). Our results show that pioglitazone can also significantly alleviate NASH in patients with prediabetes or T2DM combined with NAFLD.

In addition, pioglitazone significantly reduced plasma AST and ALT levels. Pioglitazone also significantly improved fasting glucose, HbA1c and plasma insulin levels. However, there were no significant effects on weight, BMI, total body fat, HDL or LDL compared with placebo.

Pioglitazone significantly increases the risk of adverse events, including hypoglycemia, chronic lower limb edema, atypical chest pain or epigastralgia and back or joint pain. There were two deaths in the pioglitazone arms and two in the placebo arms but insufficient power to detect an overall effect. However, because all of the studies we included were under 18 months, there is no proof so far that therapy with pioglitazone beyond 18 months brings additional histological benefits, and research in the future needs to clarify the optimal therapy duration and whether lower doses are beneficial. In addition, pioglitazone should be used with caution in T2DM patients due to the higher risk of heart failure (HF). Although a meta-analysis of 19 randomized controlled trials showed that pioglitazone treatment was associated with a reduced risk of major cardiovascular events, it was also associated with an increased risk of severe heart failure events (23).

At present, no meta-analysis has been conducted on pioglitazone in the treatment of prediabetes or T2DM patients with NAFLD. The results of a meta-analysis on NAFLD patients without T2DM showed that pioglitazone can significantly improve steatosis and inflammation but yielded significant weight gain. In addition, pioglitazone also reduced blood sugar, hemoglobin A1c, and other liver indicators, such as plasma triglycerides. This was broadly consistent with our results, except that our analysis showed no risk of weight gain, possibly due to inconsistencies in the inclusion of patients (24). Other meta-analyses also agreed on the beneficial effects of pioglitazone on lobular inflammation; however, there were still conflicting results for hepatic fibrosis (25, 26). The meta-analysis of Musso et al. showed that treatment with the antidiabetic drug pioglitazone reverses the more advanced stages of liver disease in NASH regardless of the presence of diabetes, but he combined T2DM with NAFLD and T2DM without NAFLD in a comprehensive analysis, which is still different from the study we included (27).

Recent guidelines recommend the identification of patients with advanced NAFLD with liver fibrosis and more intensive monitoring of the occurrence of complications but also acknowledge the lack of effective treatment options to reverse advanced liver disease. Our results also showed that pioglitazone was not effective in the treatment of liver fibrosis (28, 29).

### Strengths and Limitations of This Study

NAFLD is a hot spot of recent research, and there are no specific guidelines for treatment plans for NAFLD patients. Currently, there are no drugs available for the treatment of NAFLD. Although the studies we included are limited, it is the first time that we have used the gold standard of liver biopsy as the main result and used a meta-analysis to comprehensively analyze the therapeutic effects of pioglitazone on glucose metabolism and liver biological indicators in patients with NAFLD. It should be mentioned that the limitations of our meta-analysis are related to the nature of the studies included. Currently, there are few randomized controlled trials involving pioglitazone in the treatment of NAFLD and T2DM, so we conducted a comprehensive analysis of NAFLD with IGT and NAFLD with T2DM. Second, we did not conduct a separate analysis of NASH and only analyzed it as an advanced NAFLD state. Finally, the sample size of the studies we included was also small, and the duration was less than 18 months, which also affected our analysis results to some extent. However, it should be noted that the American Association for the Study of Liver Diseases and the US Food and Drug Administration have the consensus that because of the progressive characteristics of nonalcoholic fatty liver disease, it is impractical and Infeasible to conduct large-scale, long-term studies to confirm treatment-related clinical benefits (30).

## Conclusions

In conclusion, our results suggest that although pioglitazone has no significant effect on liver fibrosis, it can significantly improve liver histology, such as steatosis, inflammation and ballooning. Furthermore, pioglitazone can also improve insulin sensitivity and significantly reduce fasting blood glucose, glycosylated hemoglobin, plasma AST, ALT and other liver biological indicators. We suggest that pioglitazone can be used as a first-line therapy for NAFLD in the absence of FDA-approved pharmacological drugs for the treatment of T2DM patients with NAFLD, especially NASH.

It is also important to note that hypoglycemia, chronic lower limb edema, atypical chest pain or epigastralgia and back or joint pain are common side effects of pioglitazone treatment. In this regard, the risks and benefits should be considered before starting therapy.

In summary, due to the limitations of pioglitazone in the treatment of T2DM patients with NAFLD, such as fewer randomized controlled trials and shorter follow-up times, it is still necessary to design strict randomized controlled trials with large samples and long-term follow-up to prove the efficacy of pioglitazone to better guide clinical decision-making, patient selection and clinical practice guidelines.

## Data Availability Statement

The original contributions presented in the study are included in the article/supplementary material. Further inquiries can be directed to the corresponding author.

## Author Contributions 

JL was mainly involved in electronic retrieval, abstract screening, data extraction, data analysis and manuscript review, while JF was mainly involved in method design, electronic retrieval, abstract screening, data extraction, data analysis and interpretation and manuscript review. All authors contributed to the article and approved the submitted version.

## Funding

This research was funded by the National Natural Science Foundation of China (81670736).

## Conflict of Interest

The authors declare that the research was conducted in the absence of any commercial or financial relationships that could be construed as a potential conflict of interest.

## References

[1] Younossi Z Stepanova M Ong JP Jacobson IM Bugianesi E Duseja A . Nonalcoholic Steatohepatitis is the Fastest Growing Cause of Hepatocellular Carcinoma in Liver Transplant Candidates. Clin Gastroenterol Hepatol (2019) 17(4):748–55. doi: 10.1016/j.cgh.2018.05.057 29908364

[2] Byrne CD Targher G . NAFLD: A Multisystem Disease. J Hepatol (2015) 62(1 Suppl):S47–64. doi: 10.1016/j.jhep.2014.12.012 25920090

[3] Younossi ZM Golabi P De Avila L Paik JM Srishord M Fukui N . The Global Epidemiology of NAFLD and NASH in Patients With Type 2 Diabetes: A Systematic Review and Meta-Analysis. J Hepatol (2019) 71(4):793–801. doi: 10.1016/j.jhep.2019.06.021 31279902

[4] Sberna AL Bouillet B Rouland A Brindisi MC Nguyen A Mouillot T . European Association for the Study of the Liver (Easl), European Association for the Study of Diabetes (EASD) and European Association for the Study of Obesity (EASO) Clinical Practice Recommendations for the Management of non-Alcoholic Fatty Liver Disease: Evaluation of Their Application in People With Type 2 Diabetes. Diabetes Med (2018) 35(3):368–75. doi: 10.1111/dme.13565 29247558

[5] Jang JE Cho Y Lee BW Shin ES Lee SH . Effectiveness of Exercise Intervention in Reducing Body Weight and Glycosylated Hemoglobin Levels in Patients With Type 2 Diabetes Mellitus in Korea: A Systematic Review and Meta-Analysis. Diabetes Metab J (2019) 43(3):302–18. doi: 10.4093/dmj.2018.0062 PMC6581545 30604592

[6] Kim KS Lee BW . Beneficial Effect of Anti-Diabetic Drugs for Nonalcoholic Fatty Liver Disease. Clin Mol Hepatol (2020) 26(4):430–43. doi: 10.3350/cmh.2020.0137 PMC7641556 32791578

[7] Shah PK Mudaliar S Chang AR Aroda V Andre M Burke P . Effects of Intensive Insulin Therapy Alone and in Combination With Pioglitazone on Body Weight, Composition, Distribution and Liver Fat Content in Patients With Type 2 Diabetes. Diabetes Obes Metab (2011) 13(6):505–10. doi: 10.1111/j.1463-1326.2011.01370.x PMC3580155 21272186

[8] Le P Chaitoff A Rothberg MB McCullough A Alkhouri N . Trends in Pioglitazone Use Among U.S. Adults With Type 2 Diabetes and Suspected Nonalcoholic Fatty Liver Disease. Expert Opin Investig Drugs (2020) 29(2):205–8. doi: 10.1080/13543784.2020.1704731 31829750

[9] Bril F Biernacki DM Kalavalapalli S Lomonaco R Subbarayan SK Lai J . Role of Vitamin E for Nonalcoholic Steatohepatitis in Patients With Type 2 Diabetes: A Randomized Controlled Trial. Diabetes Care (2019) 42(8):1481–8. doi: 10.2337/dc19-0167 31332029

[10] Cusi K Orsak B Bril F Lomonaco R Hecht J Ortiz-Lopez C . Long-Term Pioglitazone Treatment for Patients With Nonalcoholic Steatohepatitis and Prediabetes or Type 2 Diabetes Mellitus: A Randomized Trial. Ann Intern Med (2016) 165(5):305–15. doi: 10.7326/M15-1774 27322798

[11] Belfort R Harrison SA Brown K Darland C Finch J Hardies J . A Placebo-Controlled Trial of Pioglitazone in Subjects With Nonalcoholic Steatohepatitis. N Engl J Med (2006) 355(22):2297–307. doi: 10.1056/NEJMoa060326 17135584

[12] Yan HM Xia MF Wang Y Chang XX Yao XZ Rao SX . Efficacy of Berberine in Patients With Non-Alcoholic Fatty Liver Disease. PloS One (2015) 10(8):e0134172. doi: 10.1371/journal.pone.0134172 26252777 PMC4529214

[13] Williamson RM Price JF Glancy S Perry E Nee LD Hayes PC . Prevalence of and Risk Factors for Hepatic Steatosis and Nonalcoholic Fatty Liver Disease in People With Type 2 Diabetes: The Edinburgh Type 2 Diabetes Study. Diabetes Care (2011) 34(5):1139–44. doi: 10.2337/dc10-2229 PMC3114489 21478462

[14] Leite NC Salles GF Araujo AL Villela-Nogueira CA Cardoso CR . Prevalence and Associated Factors of non-Alcoholic Fatty Liver Disease in Patients With Type-2 Diabetes Mellitus. Liver Int (2009) 29(1):113–9. doi: 10.1111/j.1478-3231.2008.01718.x 18384521

[15] Kotronen A Juurinen L Hakkarainen A Westerbacka J Cornér A Bergholm R . Liver Fat is Increased in Type 2 Diabetic Patients and Underestimated by Serum Alanine Aminotransferase Compared With Equally Obese Nondiabetic Subjects. Diabetes Care (2008) 31(1):165–9. doi: 10.2337/dc07-1463 17934148

[16] Wild SH Walker JJ Morling JR Westerbacka J Cornér A Bergholm R . Cardiovascular Disease, Cancer, and Mortality Among People With Type 2 Diabetes and Alcoholic or Nonalcoholic Fatty Liver Disease Hospital Admission. Diabetes Care (2018) 41(2):341–7. doi: 10.2337/dc17-1590 29167212

[17] Stepanova M Rafiq N Makhlouf H Agrawal R Kaur I Younoszai Z . Predictors of All-Cause Mortality and Liver-Related Mortality in Patients With non-Alcoholic Fatty Liver Disease (NAFLD). Dig Dis Sci (2013) 58(10):3017–23. doi: 10.1007/s10620-013-2743-5 23775317

[18] Xia MF Bian H Gao X . NAFLD and Diabetes: Two Sides of the Same Coin? Rationale for Gene-Based Personalized NAFLD Treatment. Front Pharmacol (2019) 10:877. doi: 10.3389/fphar.2019.00877 31447675 PMC6691129

[19] Lee YH Cho Y Lee BW Park CY Lee DH Cha BS . Nonalcoholic Fatty Liver Disease in Diabetes. Part I: Epidemiology and Diagnosis. Diabetes Metab J (2019) 43(1):31–45. doi: 10.4093/dmj.2019.0011 30793550 PMC6387876

[20] Portillo-Sanchez P Bril F Maximos M Lomonaco R Biernacki D Orsak B . High Prevalence of Nonalcoholic Fatty Liver Disease in Patients With Type 2 Diabetes Mellitus and Normal Plasma Aminotransferase Levels. J Clin Endocrinol Metab (2015) 100(6):2231–8. doi: 10.1210/jc.2015-1966 PMC6287506 25885947

[21] Maximos M Bril F Portillo Sanchez P Lomonaco R Orsak B Biernacki D . The Role of Liver Fat and Insulin Resistance as Determinants of Plasma Aminotransferase Elevation in Nonalcoholic Fatty Liver Disease. Hepatology (2015) 61(1):153–60. doi: 10.1002/hep.27395 25145475

[22] Leite NC Villela-Nogueira CA Pannain VL Bottino AC Rezende GF Cardoso CR . Histopathological Stages of Nonalcoholic Fatty Liver Disease in Type 2 Diabetes: Prevalences and Correlated Factors. Liver Int (2011) 31(5):700–6. doi: 10.1111/j.1478-3231.2011.02482.x 21457442

[23] Lincoff AM Wolski K Nicholls SJ Nissen SE . Pioglitazone and Risk of Cardiovascular Events in Patients With Type 2 Diabetes Mellitus: A Meta-Analysis of Randomized Trials. JAMA (2007) 298(10):1180–8. doi: 10.1001/jama.298.10.1180 17848652

[24] Musso G Gambino R Cassader M Pagano G . A Meta-Analysis of Randomized Trials for the Treatment of Nonalcoholic Fatty Liver Disease. Hepatology (2010) 52(1):79–104. doi: 10.1002/hep.23623 20578268

[25] Singh S Khera R Allen AM Murad MH Loomba R . Comparative Effectiveness of Pharmacological Interventions for Nonalcoholic Steatohepatitis: A Systematic Review and Network Meta-Analysis. Hepatology (2015) 62(5):1417–32. doi: 10.1002/hep.27999 26189925

[26] He L Liu X Wang L Yang Z . Thiazolidinediones for Nonalcoholic Steatohepatitis: A Meta-Analysis of Randomized Clinical Trials. Med (Baltimore) (2016) 95(42):e4947. doi: 10.1097/MD.0000000000004947 PMC5079311 27759627

[27] Musso G Cassader M Paschetta E Gambino R . Thiazolidinediones and Advanced Liver Fibrosis in Nonalcoholic Steatohepatitis: A Meta-Analysis. JAMA Intern Med (2017) 177(5):633–40. doi: 10.1001/jamainternmed.2016.9607 PMC5470366 28241279

[28] European Association for the Study of The L European Association for the Study Of D European Association for the Study Of O . Easl-Easd-Easo Clinical Practice Guidelines for the Management of non-Alcoholic Fatty Liver Disease. Diabetologia (2016) 59(6):1121–40. doi: 10.1007/s00125-016-3902-y 27053230

[29] Glen J Floros L Day C Pryke R . Non-Alcoholic Fatty Liver Disease (NAFLD): Summary of NICE Guidance. BMJ (2016) 354:i4428. doi: 10.1136/bmj.i4428 27605111

[30] Sanyal AJ Friedman SL Mccullough AJ Dimick-Santos L . Challenges and Opportunities in Drug and Biomarker Development for Nonalcoholic Steatohepatitis: Findings and Recommendations From an American Association for the Study of Liver Diseases-U.s. Food and Drug Administration Joint Workshop. Hepatology (2015) 61(4):1392–405. doi: 10.1002/hep.27678 PMC4900161 25557690

