# Corrigendum: Pioglitazone for NAFLD Patients With Prediabetes or Type 2 Diabetes Mellitus: A Meta-Analysis

**DOI:** 10.3389/fendo.2022.840299

**Published:** 2022-02-16

**Authors:** Jingxuan Lian, Jianfang Fu

**Affiliations:** Department of Endocrinology, Xijing Hospital of Air Force Medical University, Xi’an, China

**Keywords:** NAFLD, type 2 diabetes mellitus, pioglitazone, meta-analysis, prediabetes

In the article as originally published the labeling of the forest plots shown in **Figures 3A-F** were not correct. The use and positioning of the text “favours [pioglitazone]” and “favours [placebo]” seemed to imply that the placebo group had a higher risk of morbidity. A correct version of the **Figure 3**, with updated labeling is shown here.

**Figure 3:**
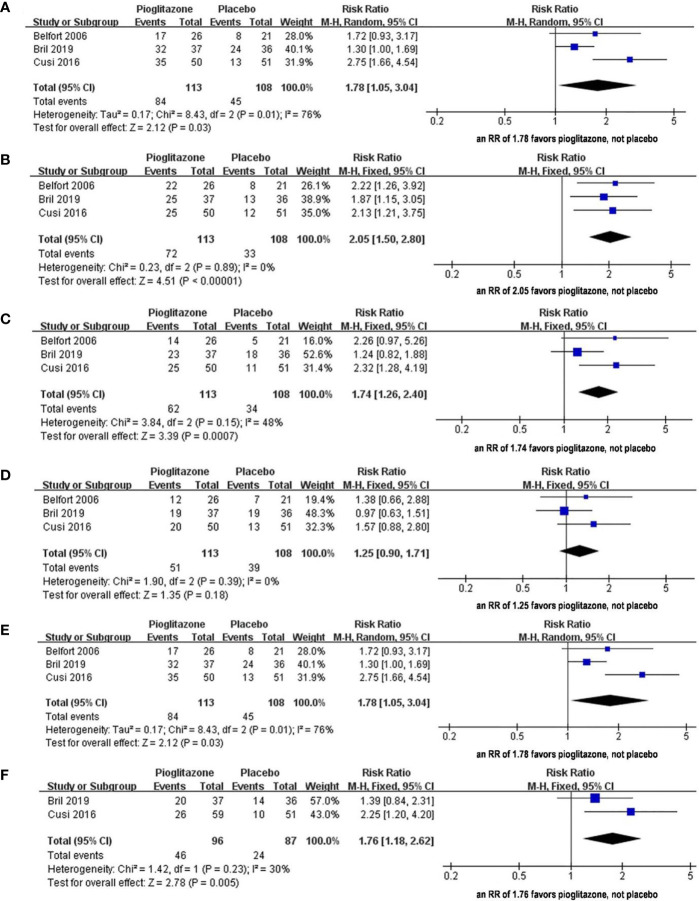
The effect of pioglitazone in hepatic histologic scores (steatosis grade) with improvement of at least 1 grade **(A)**. The effect of pioglitazone in hepatic histologic scores (inflammation grade) with improvement of at least 1 grade **(B)**. The effect of pioglitazone in hepatic histologic scores (ballooning grade) with improvement of at least 1 grade **(C)**. The effect of pioglitazone in hepatic histologic scores (fibrosis stage) with improvement of at least 1 grade **(D)**. The effect of pioglitazone in resolution of NASH without worsening of fibrosis **(E)**. The effect of pioglitazone in reduction of at least 2 points in hepatic histologic scores (from two different parameters) **(F)**.

The authors apologize for this error and state that this does not change the scientific conclusions of the article in any way. The original article has been updated.

## Publisher’s Note

All claims expressed in this article are solely those of the authors and do not necessarily represent those of their affiliated organizations, or those of the publisher, the editors and the reviewers. Any product that may be evaluated in this article, or claim that may be made by its manufacturer, is not guaranteed or endorsed by the publisher.

